# Human placental extract activates a wide array of gene expressions related to skin functions

**DOI:** 10.1038/s41598-022-15270-y

**Published:** 2022-06-30

**Authors:** Phooi-Yee Chang, Lit-Chein Chin, Koichi Kimura, Yasukazu Nakahata

**Affiliations:** 1 Department of Research & Development, Melsmon Pharmaceutical Co. Ltd., Tokyo, 171-0014 Japan; 2 Department of Neurobiology & Behavior, Nagasaki University Graduate School of Biomedical Sciences, Nagasaki, 852-8523 Japan

**Keywords:** Molecular biology, Diseases, Molecular medicine

## Abstract

As skin aging is one of the most common dermatological concerns in recent years, scientific research has promoted treatment strategies aimed at preventing or reversing skin aging. Breakdown of the extracellular matrix (ECM), such as collagen and elastin fibers, in the skin results in decreased skin elasticity and tension. Cutaneous cells, especially fibroblasts in the dermis layer of the skin, mainly produce ECM proteins. Although clinical studies have demonstrated that placental extract (PE) has positive effects on skin health, the molecular mechanisms by which PE acts against skin aging are still largely unknown. In this study, we performed RNA-sequence analysis to investigate whether human PE (HPE) alters ECM-related gene expression in normal human dermal fibroblast (NHDF) cells. Gene ontology analysis showed that genes related to extracellular matrix/structure organization, such as *COL1A1*, *COL5A3*, *ELN*, and *HAS2* were highly enriched, and most of these genes were upregulated. We further confirmed that the HPE increased the type I collagen, proteoglycan versican, elastin, and hyaluronan levels in NHDF cells. Our results demonstrate that HPE activates global ECM-related gene expression in NHDF cells, which accounts for the clinical evidence that the HPE affects skin aging.

## Introduction

Skin aging has become one of the most common dermatological concerns recently as the world’s population is growing older^1^ and is triggered by both intrinsic and extrinsic causes. Intrinsic skin aging occurs naturally with chronological aging, which is mainly influenced by genetic predisposition, neuroendocrinology, and metabolic factors acting in an age-dependent manner, and it also can be accelerated by external environment factors such as ultraviolet radiation (UVR), environmental pollutants, smoking, low quality nutrition, and microbial infections^2–6^. However, skin has the ability to protect itself from various external stressors through the neuroendocrine systems by producing several protective molecules, including vitamin D and melatonin^7–11^. Both intrinsic and extrinsic skin aging induce the breakdown of extracellular matrix (ECM) proteins in the skin, resulting in decreased skin elasticity and tension. Fibroblasts in the dermis layer of the skin has been shown to maintain the skin’s strength, elasticity, and moisture by producing ECM proteins such as collagen fibrils and the elastic fibers^12,13^. It is worthy to note that senescent fibroblasts appear to accumulate in the skin with age^14,15^, and the elimination of senescent fibroblasts in the skin has recently been demonstrated to attenuate intrinsic skin aging^16^. The hallmarks of fibroblast aging include genome instability, telomere attrition, epigenetic alterations, mitochondrial dysfunction, cellular senescence, altered intercellular communication, and loss of proteostatis^17^.

Strategies against skin aging have been developed since ancient times. Herbal products, including botanicals such as aloin, ginsenoside, and curcumin, have long been used as skincare cosmetics^18^. Recent research has provided molecular evidence on how botanicals act against skin aging^18,19^. In addition to herbal products, minerals, vitamins and their chemical compound derivatives have been scientifically revealed to possess potential against skin aging^20,21^. Placental extract (PE), which is derived from mammalian tissues, is also known to have positive effects on skin health^22,23^. PE has originally attracted attention worldwide due to its therapeutic potential in several medical applications^22,24–28^, such as relieving the symptoms caused by menopause^29–31^. Recent studies have shown that porcine PE enhances type I collagen production and cell proliferation of the human osteoblastic cell line Saos-2 and human dermal fibroblasts^32,33^. Human PE (HPE) has also been shown to increase the production of type I collagen in human gingival fibroblasts^34^. Currently, HPE is approved for subcutaneous injection as prescription drug for treatment of menopausal disorder in Japan. However, molecular investigations of PE are limited compared to the clinical studies of PE. Moreover, comprehensive gene expression analyses of the effects of PE on dermal fibroblasts remain largely unknown.

In this study, we performed the RNA sequencing (RNA-seq) and gene ontology (GO) analyses to uncover the effects of HPE on normal human dermal fibroblasts (NHDF). We demonstrated by GO enrichment analyses that the top scores of biological process, molecular function, and cellular component were ‘extracellular matrix organization’, ‘collagen binding’, and ‘extracellular matrix’, respectively, all of which are involved in skin functions.

## Results

### Effects of HPE on type I collagen productions at mRNA and protein levels

**Figure 1:**
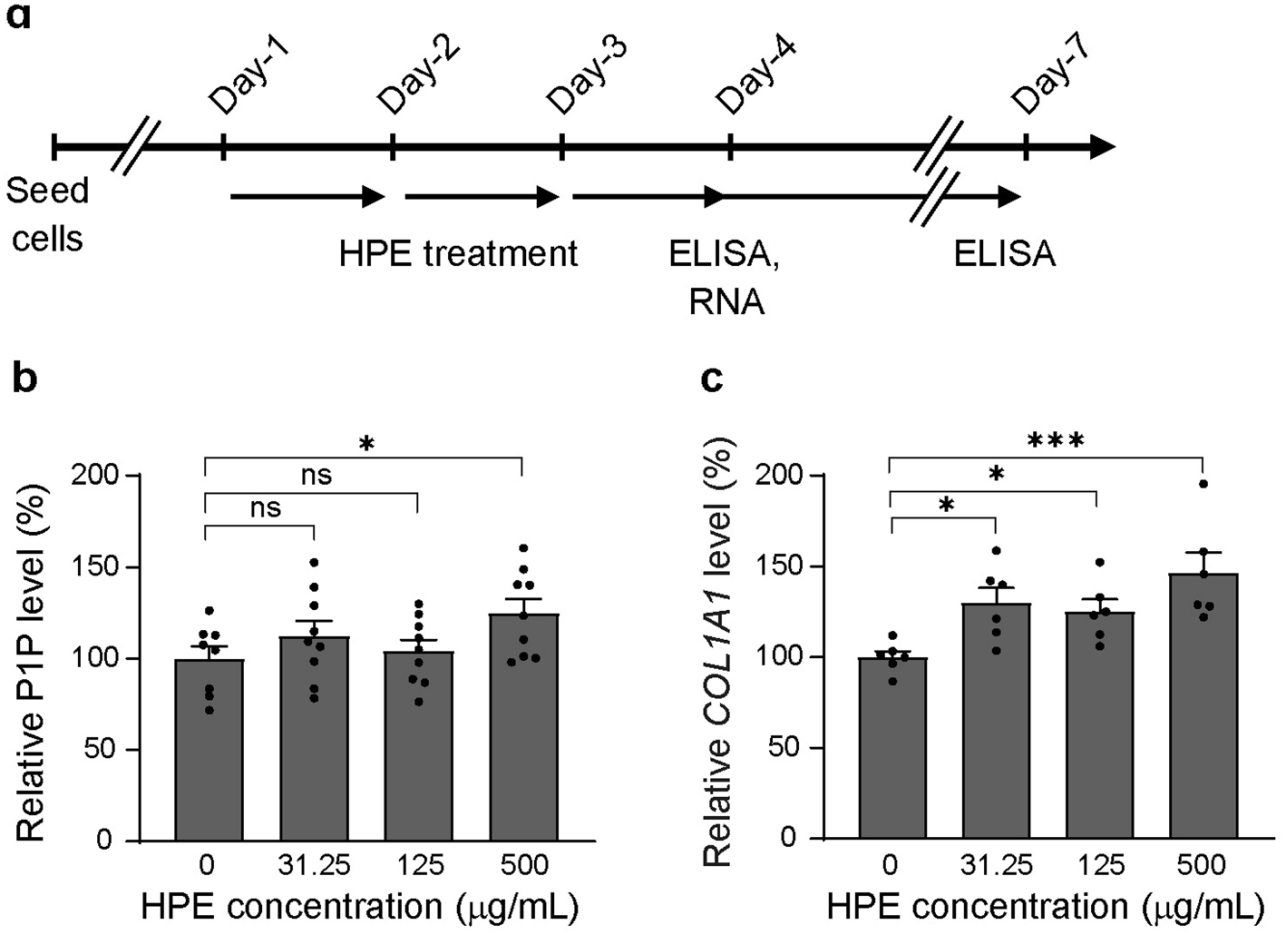
HPE increases collagen levels both at mRNA and protein levels. (**a**) Schematic diagram of HPE treatment procedure. **(b** and **c**) Effects of HPE on PIP production and COL1A1 mRNA expression in cultured NHDF cells. Cells were treated with or without HPE for 72 h. *p < 0.05, ***p < 0.001, or ns, not significant, compared to the control, 0 mg/mL HPE, by ANOVA followed by Fisher’s LSD test.

Type I collagen is mainly produced by dermal fibroblasts and is the most abundant structural protein in the skin. Therefore, regulation of type I collagen expression is important for maintaining the skin under good conditions. To investigate the effects of human placental extract (HPE) on type I collagen synthesis in NHDF cells, we performed a procollagen type I peptide (PIP) ELISA assay. After the administration of HPE at different concentrations to the NHDF cells, we measured the amount of PIP in the culture supernatants. The PIP levels increased significantly after 72 h incubation with 500 µg/mL of HPE (Fig. 1b), which is consistent with the results of previous studies that used porcine placental extract^32,33^. We further investigated the effects of HPE on type I collagen gene expression levels by using qPCR. Type I collagen is composed of two α1 chains and one α2 chain which are encoded by *COL1A1* and *COL1A2* genes, respectively. We found that HPE significantly increased the expression level of *COL1A1* mRNA, but not *COL1A2* mRNA, in the NHDF cells (Fig. 1c, Supplementary Fig. S1). The *COL1A1* mRNA levels were increased by HPE treatment in a dose-dependent manner, and the expression level of 500 µg/mL HPE-treated cells was approximately 1.4-fold higher than that of the untreated cells. We have also tested NHDF cell lines from different donors and found that HPE could stimulate the COL1A1 protein and mRNA levels in 2 out of 3 of the NHDF cell lines tested (Supplementary Fig. S2). Taken together, we confirmed that HPE treatment significantly increased type I collagen production at the protein level, as previously reported^34^. Furthermore, we demonstrated that HPE increased the expression of *COL1A1* mRNA in NHDF cells. Further experiments were performed using the NHDF cell line from Cell Applications Lot 2485.

### Differentially expressed genes (DEGs) analysis

**Figure 2:**
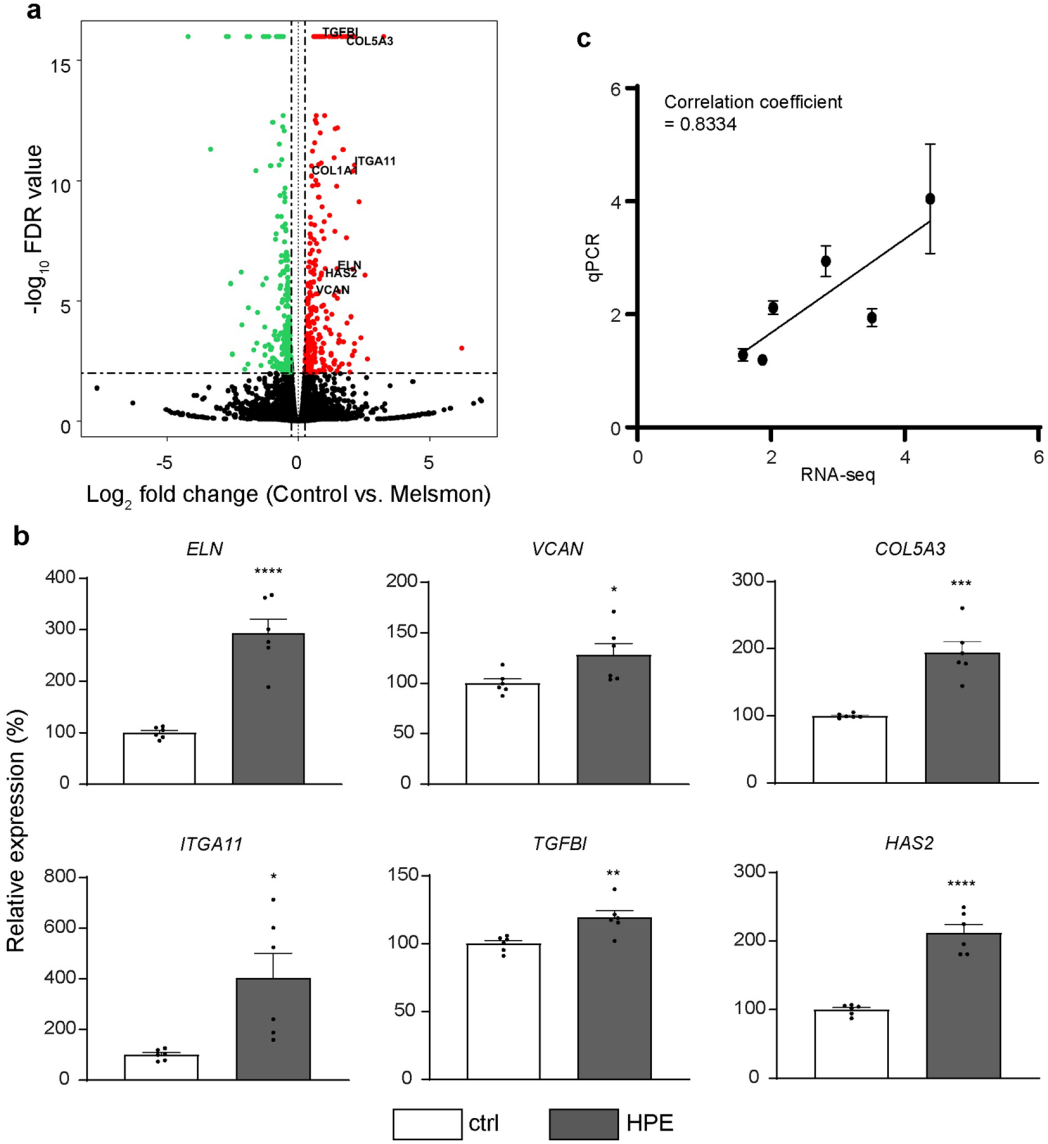
HPE globally affects gene expression levels in NHDF cells. (**a**) Volcano plot of differentially expressed genes of HPE-treated NHDF cells. Red, black, and green dots represent upregulated, non-differentially expressed, and down-regulated genes, respectively. (**b**) Six selected genes from DEGs were validated by qPCR. GAPDH gene was used as the reference. White or black bars indicate control (ctrl) or HPE-treated samples, respectively. *p < 0.05, **p < 0.01, or ***p < 0.001, ****p < 0.0001 compared to the control sample by Unpaired Student’s *t*-test. (c) The levels of 6 selected genes show the correlation between the results of RNA-seq and qPCR (correlation coefficient = 0.8334).

The increases in *COL1A1* mRNA expression in NHDF cells prompted us to investigate whether other genes involved in skin functions are also activated by HPE. To that end, we performed RNA-sequencing (RNA-seq) using NHDF cells treated with or without 500 µg/mL HPE for 72 h. The significantly differentially expressed genes (DEGs) of the HPE-treated NHDF cells were defined by an FDR p-value of < 0.01 and fold change of > 1.2. The gene expression profiles of the treated cells were analyzed, and the expression patterns were represented by volcano plot (Fig. 2a), which shows the DEGs in the treated cells; the green dots represent the significantly downregulated DEGs, whereas the red dots represent significantly upregulated genes. The results revealed that out of 504 DEGs, 271 genes were upregulated and 233 genes were downregulated.

We then selected six ECM-related DEGs (*ELN*, *COL5A3*, *ITGA11*, *VCAN*, *TGFBI*, and *HAS2*) to validate the RNA-seq data by qPCR. The qPCR results demonstrated that HPE triggered 2.9- and 1.3-fold increases in elastin (*ELN*) and proteoglycan versican (*VCAN*) transcripts, respectively (Fig. 2b), which are some of the core ECM components found in human skin^35^. We also confirmed that HPE significantly increased the expression of *COL5A3*, *ITGA11*, *TGFBI*, and *HAS2* by 1.9-, 4-, 1.2-, and 2.1-fold respectively (Fig. 2b). We then calculated the correlation coefficient between RNA-seq and qPCR results and showed a highly positive correlation (*p* = 0.83) (Fig. 2c),validating the RNA-seq data for further analyses.

### Gene ontology (GO) enrichment analysis revealed that the HPE alters gene expressions positively according to the skin function

**Figure 3:**
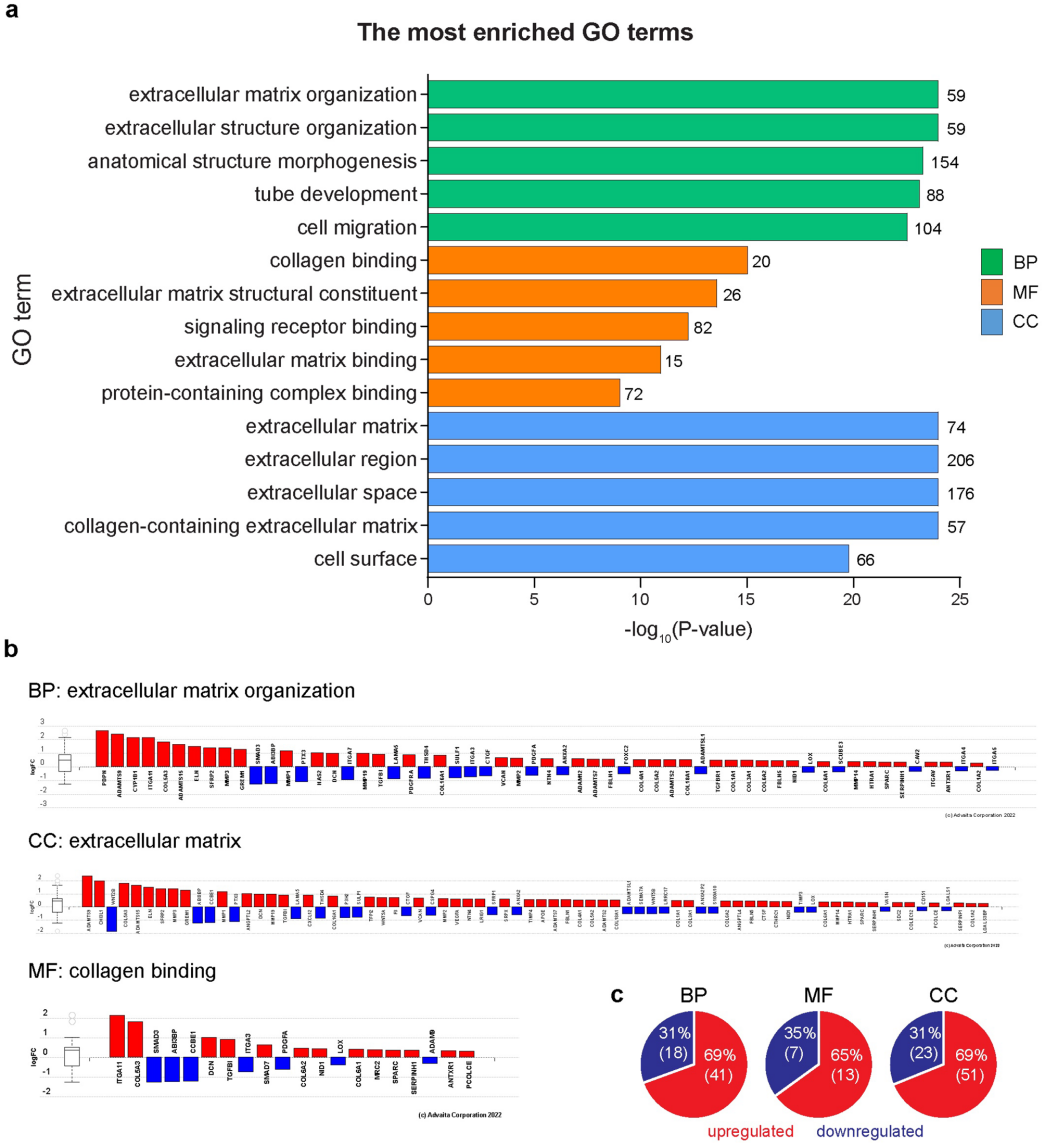
Gene ontology (GO) enrichment analyses revealed ECM enhancing functions by HPE. (**a**) Top 5 significantly enriched GO terms in the BP (green), MF (orange), and CC (blue). (**b**) DEGs under the top identified BP, MF, and CC. Red and blue bars represent up and downregulated genes, respectively. (**c**) Pie charts show percentages and numbers of upregulated (red) and downregulated (blue) DEGs in BP, MF, and CC. BP: Biological process; MF: Molecular function; CC: Cellular components.

In the HPE-treated NHDF cells, GO enrichment analysis categorized the 504 DEGs into three major categories: biological process (BP), molecular function (MF), and cellular component (CC) (Fig. 3a and Supplementary Table S1). Among these DEGs, a significant number of DEGs were found to be related to the ECM. The top five most significant BP GO terms were ‘extracellular matrix organization’, ‘extracellular structure organization’, ‘anatomical structure morphogenesis’, ‘tube development’, and ‘cell migration’. Forty-one (69%) out of 59 DEGs belonging to ‘extracellular matrix organization’ were upregulated (Fig. 3b and c). As for MF, the top five most significant GO terms were ‘collagen binding’, ‘extracellular matrix constituent’, ‘signaling receptor binding’, ‘extracellular matrix binding’, and ‘protein-containing complex binding’. Among the 20 DEGs related to ‘collagen binding’, 13 (65%) were upregulated (Fig. 3b and c). Finally, for the CC category, the top five most significant GO terms were ‘extracellular matrix’, ‘extracellular region’, and ‘extracellular space’, ‘collagen-containing extracellular matrix’, and ‘cell surface’. Among the 74 DEGs related to the ‘extracellular matrix’, 51 (69%) were upregulated (Fig. 3b and c). Taken together, GO enrichment analyses revealed that HPE treatment of NHDF cells predominantly altered ECM-related genes, most of which were upregulated, strongly suggesting that HPE treatment ameliorates skin functions in vivo.

### The HPE induced ECM-related proteins

**Figure 4:**
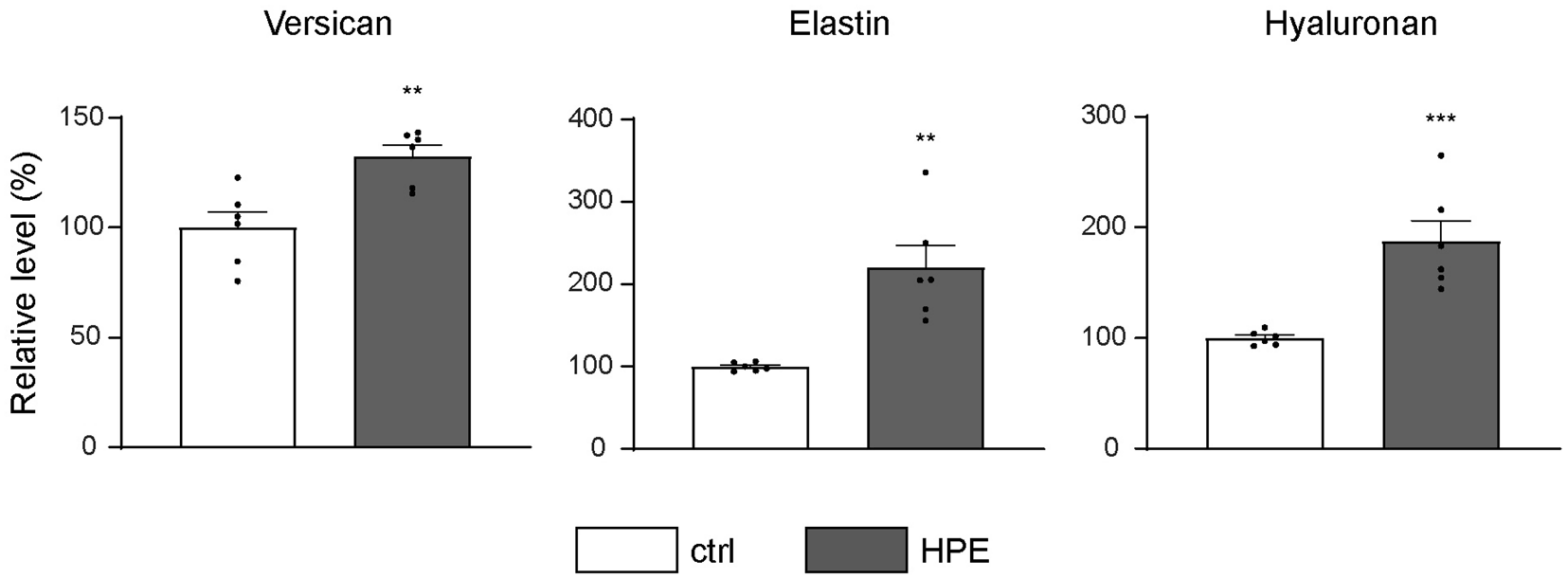
HPE affects the production of ECM components in NHDF cells. The amount of proteoglycan versican, elastin and hyaluronan in culture supernatants of NHDF cells after HPE treatment were measured by ELISA. White or black bars indicate control (ctrl) or HPE-treated samples, respectively. *p < 0.05, **p < 0.01, or ***p < 0.001, ****p < 0.0001 compared to the control sample by unpaired Student’s *t*-test.

As we showed that HPE increased type I collagen at the protein level (Fig. 1b), we performed ELISA assays for versican, elastin, and hyaluronan (HA), in which gene expression was increased by HPE treatment. Versican is a large extracellular matrix proteoglycan translated from *VCAN* mRNA, and hyaluronan is synthesized by HAS2. HPE treatment at 500 µg/mL significantly increased all tested ECM-related proteins (Fig. 4). These results imply that not only type I collagen, versican, elastin, and hyaluronan, but also many other ECM-related proteins, are probably increased by the HPE treatment.

In conclusion, we propose that HPE contributes against skin aging by activating global ECM-related gene expression in dermal fibroblasts in vivo.

## Discussion

Aging and environmental stress can impair the quality of the ECM and consequently affect the overall skin functions. To maintain the structural integrity and elasticity of the skin, it is necessary to regulate the functions of dermal fibroblasts and the subsequent ECM protein levels. Previous studies have reported that the porcine and human placental extracts (PPE and HPE, respectively) promote type I collagen production in human cell cultures^32–34^. In this study, we found that type I collagen production in NHDF cells was enhanced after 72 h of treatment with 500 µg/mL HPE (Fig. 1a). The expression level of *COL1A1* mRNA is also increased significantly after HPE treatment, as shown in Fig. 1b. There are 28 known types of collagen in the human body^36^. Although collagen types I and II are the most abundant, other minor collagens, which account for less than 10% of the total collagen content, also have important roles in collagenous tissues. Based on our DEGs analysis, the HPE not only affected the type I collagen gene expression, but also upregulated the other types of collagen coding genes such as *COL5A3*, *COL16A1*, *COL4A1*, *COL5A2*, *COL18A1*, *COL3A1*, *COL6A1*, and *COL6A2* (Supplementary Table S2). This finding further supports that the HPE can improve the skin integrity by upregulating collagen. We further showed that HPE dominantly increased the expression of a wide array of genes related to skin functions. Furthermore, we showed that some ECM proteins were increased by HPE administration in NHDF cells. These findings therefore suggest that HPE contributes against skin aging by activating global ECM-related gene expression in dermal fibroblasts in vivo.

Elastic fibers are a major component of the dermal ECM and consist of two distinct components. The major component is the elastin fiber, which is composed of crosslinked tropoelastin, while the other is fibrillin-rich microfibrils^13,37^. In this study, HPE treatment significantly increased the *elastin* mRNA expression level (Fig. 2b) in NHDF cells, suggesting that the HPE improves skin elasticity and flexibility through the upregulation of elastin synthesis. In contrast, HPE-treated NHDF cells showed a 1.4-fold increase in *fibulin-5* (*FBLN5*) mRNA levels (*p* = 1.00E-06, Supplementary Dataset S1). Fibulin-5, a component of microfibrils, accelerates elastic fiber assembly in dermal fibroblasts^38^. Thus, the increase in *FBLN5* mRNA levels in HPE-treated cells suggests that HPE enhances elastic fiber assembly in the cells. Taken together, our results suggest that HPE promotes elastin synthesis and elastic fiber formation in human dermal fibroblasts.

In addition to fibrous proteins such as collagens and elastin, ECM is also composed of nonfiber-forming molecules, which are mainly proteoglycans (PGs) and hyaluronan (HA). There are two groups of matrix PGs. The first group is a family of small leucine-rich PGs (SLRPs) including decorin, biglycan, fibromodulin, lumican and others, while the second family of matrix PGs is the hyalectan family of large chondroitin sulfate (CS) PGs, which include versican and aggrecan. PGs belonging to the hyalectan family contain an HA-binding N-terminal domain and a lectin-binding C-terminal domain^39^. Versican is largely distributed throughout the body and is part of the ECM in several tissues. Versican appears to be associated with elastic fibers and it plays a role in controlling cell adhesion, migration, proliferation, and ECM assembly^40,41^. Versican regulates the cell and tissue behaviors through interactions with various ECM proteins, such as collagen I, fibronectin, and fibulins^42^. In addition, versican interacts with HA to create a pericellular matrix that facilitates cell sorting, proliferation, migration, and survival^43^. In this study, we found that HPE significantly increased the expression of both versican (*VCAN*) and hyaluronan synthase 2 (*HAS2*) transcripts in NHDF cells. Furthermore, we demonstrated that versican and HA levels were significantly increased after HPE treatment (Fig. 4), suggesting that HPE might help to improve skin health by modulating cellular processes.

Excessive collagen production is associated with fibrosis development, which is triggered by TGF-β and other cytokines. However, the ECM profiles of dermal fibroblast skin fibrosis model^44^ and animal lung fibrosis models^45,46^ are very much different than what we observed in our study, such as strong stimulation of type-I collagen mRNA expression of up to tenfold, as opposed to ~ 1.4-fold in our study (Fig. 1c, Supplementary table 2). In addition, the simultaneous moderate stimulation of HA production, versican proteoglycan, and other ECM components in NHDF suggests that HPE treatment has overall beneficial effects on maintaining skin health.

The procedure to manufacture the HPE used in this study includes the hydrolysis of human placenta from healthy donors with acid, neutralized to pH 6.8–7.0, and sterilized at 121 °C for 30 min. The resulting HPE contains a wide array of amino acids, nucleic acids, sugars, and lipids^29,47^. Gel filtration chromatography showed that HPE was separated into six peaks, ranging from 200 to 1300 Da (data not shown). Although we did not identify significant factors, or even fractions, which can trigger gene expression identified in this study, investigations to identify the factors will be indispensable to reveal the molecular mechanisms of the anti-aging effects on skin induced by HPE.

NHDF cells used in this study were derived from a 66-year-old Caucasian woman; however, the individual population doubling levels of the cells used in this study were between 4 and 2. This means that the cells were still in the proliferative phase, not in the senescent phase. Therefore, the beneficial effect of HPE treatment for improving skin health can be expected not only for middle-aged people, but also for young people. In addition, it would be interesting to investigate whether our findings could be expanded to senescent NHDF cells, namely elderly people.

## Materials and methods

### Human placental extract

Human placental extract (HPE) from Melsmon Pharmaceutical Co., Ltd., Japan was used in this study^29^. This HPE contained 50 mg/mL of human placental hydrolysate.

### Human dermal fibroblasts and culture conditions

NHDF cells (Cat# 106-05a; Lot No. 2485; normal human facial skin from a 66 years old Caucasian female) and culture ingredients were purchased from Cell Application, Inc., USA. The cells were cultured in an endothelial basal medium containing 3% endothelial cell growth supplement at 37 °C with 5% CO_2_. Cells in this study were between 7 to 9 passages, corresponding to individual population doubling levels between 4 and 2.

### Enzyme-linked immunosorbent assay (ELISA)

Procollagen type I peptide (PIP) and proteoglycan versican (VCAN) levels were measured using a Procollagen Type I C-Peptide EIA kit (TaKaRa, Japan) and Human VCAN (Versican/PG-M) ELISA kit (Elabscience, USA), respectively. A total of 3 × 10^4^ cells were seeded per well in a 6-well plate and treated with or without HPE at 80% confluency for 3 days, and the culture medium was replaced every 24 h (Fig. 1a). The amount of culture media applied to each well in 6-well plate is 2 mL unless otherwise specified. The culture supernatants were collected and measured using ELISA kits, following the manufacturer’s instructions.

Elastin (ELN) and hyaluronan (HA) levels were measured using the Human Elastin SimpleStep ELISA kit (abcam, UK) and Hyaluronan Quantikine ELISA kit (R&D Systems, USA), respectively. A total of 5 × 10^4^ cells were seeded per well in a 6-well plate and treated with or without HPE at 80% confluency for 2 days, and the culture medium (2 mL) was replaced every 24 h. On the third day, the culture medium was replaced with 1 mL of culture medium with or without HPE and incubated for another 96 h. The culture supernatants were collected and measured using ELISA kits, following the manufacturer’s instructions.

### Quantitative PCR (qPCR) analysis

Total RNA was purified from cultured cells using Sepasol-RNA I Super G (Nacalai Tesque, Japan) following the manufacturer's protocol. First-strand cDNA synthesis was performed using SuperScript II reverse transcriptase (Invitrogen, USA) with random primers. Quantitative PCR was performed in the presence of KAPA SYBR FAST Universal 2X qPCR Master Mix (Nippon Genetics, Japan) on a Thermal Cycler Dice Real Time System III (Takara Bio, Japan) under the following conditions: denaturation at 95 °C for 3 min, followed by 40 cycles at 95 °C for 3 s and 60 °C for 20 s. The primer sets used in this study are listed in Supplementary Table S3. GAPDH gene was used as a reference gene for data normalization.

### RNA-seq

Total RNA was extracted from six samples (three biological replicates of HPE-treated NHDF and three biological replicates of control) using the RNAeasy Plus Mini Kit (Qiagen, Germany) according to the manufacturer's instructions. The quality and quantity of the extracted RNA were determined using the Qubit® RNA BR Assay Kit (Invitrogen, USA). The libraries were prepared using AmpliSeq cDNA Synthesis for Illumina Kit (Illumina, USA), AmpliSeq for Illumina Transcriptome Human Gene Expression Panel (Illumina, USA), AmpliSeq Library PLUS for Illumina (Illumina, USA), and AmpliSeq CD Indexes for Illumina (Illumina, USA), according to the manufacturer’s instructions. The quality and quantity of the libraries were checked using an Agilent 2100 Bioanalyzer (Agilent Technologies, USA) and the libraries were diluted and pooled according to the manufacturer’s recommendations and sequenced using the MiSeq® system (Illumina, USA). RNA-seq data were deposited in the DNA Data Bank of Japan (DDBJ) Sequence Read Archive (DRA) (accession no. E-GEAD-469).

### Differential expressed genes (DEG) identification and Gene ontology (GO) analysis

The sequence file obtained in FASTQ format was imported into the CLC Genomics Workbench (Version 20.0.4). The read counts of each sample were processed using the RNA-seq analysis tool and mapped to the reference human genome hg19 using default parameter settings. Differential expression was analyzed using the differential expression tool. Differentially expressed genes (DEGs) were identified by filtering with false discovery rate (FDR) *p*-value of < 0.01 and fold change of > 1.2. GO enrichment analysis was performed using the iPathwayGuide software (https://ipathwayguide.advaitabio.com/)^48^.

### Statistical analysis

Values are reported as mean ± SEM. Statistical differences were determined by one-way analysis of variance (ANOVA) followed by Fisher’s LSD test or unpaired Student’s *t*-test. GraphPad Prism Software (ver.8) was used to perform statistical analyses and create graphs. Statistical significance is displayed as * (*p* < 0.05), ** (*p* < 0.01), *** (*p* < 0.001) or **** (*p* < 0.0001).

## Supplementary Information

Supplementary Information 1.

Supplementary Information 2.

## Data Availability

The dataset generated in this study is available in the GEA (Genomic Expression Archive) repository [(E-GEAD-469) (https://ddbj.nig.ac.jp/public/ddbj_database/gea/experiment/E-GEAD-000/E-GEAD-469/)] and from the corresponding author on request.
