# Supplementary material for: Human placental extract activates a wide array of gene expressions related to skin functions
Source: Sci Rep. 2022 Jun 30;12:11031. doi: 10.1038/s41598-022-15270-y (PMC9246867; doi:10.1038/s41598-022-15270-y)
Supplement: Supplementary file 1 — Supplementary Information 1. [file 41598_2022_15270_MOESM1_ESM.pdf]

Supplementary information

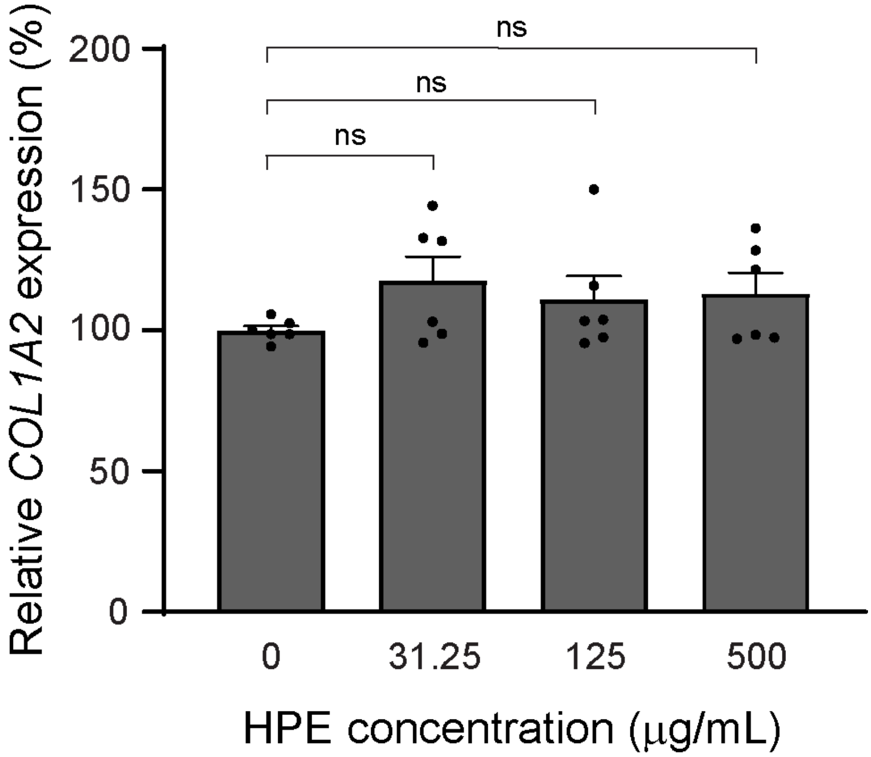

Supplementary Fig. S1. The HPE had no effect on *COL1A2* mRNA expression in NHDF cells. Cells were treated with or without the HPE for 72 h. The GAPDH gene was used as reference. ns, not significant, compared to the control, 0 mg/ml HPE, by ANOVA followed by Fisher's LSD test.

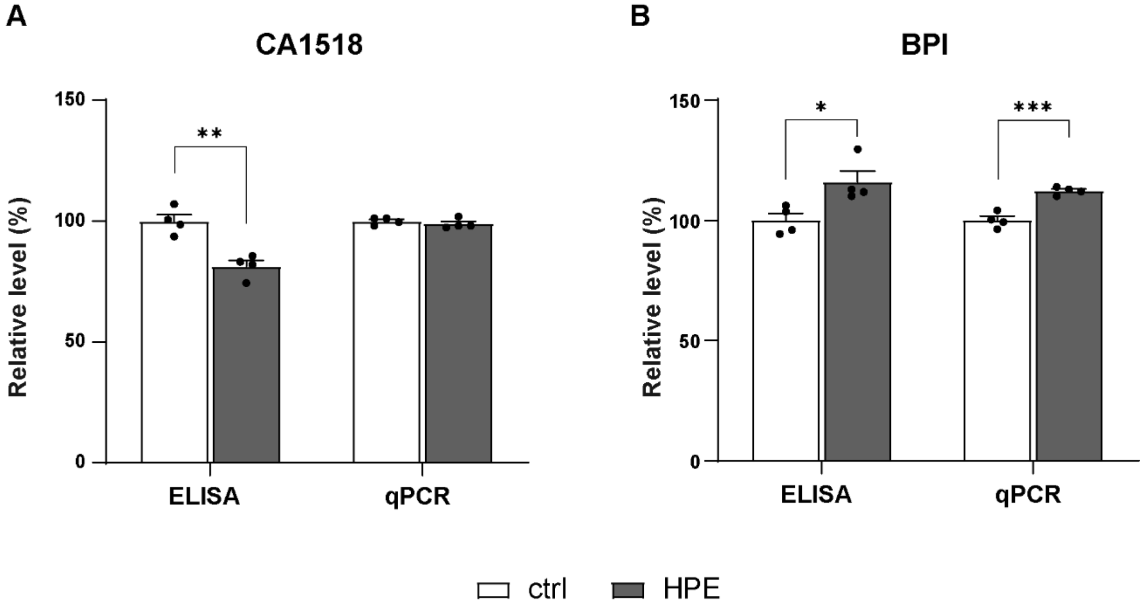

Supplementary Fig S2. The effect of HPE on the COL1A1 protein and mRNA levels in different NHDF cell lines. (a) Cell Applications (Cat# 106-05a) Lot No. 1518; normal human dermal fibroblasts from facial skin of 63 years old Caucasian female. (b) Biopredic International (Cat# FIB101) Batch No. FIB101035; normal human dermal fibroblasts from abdomen skin of 30 years old Caucasian female.

Supplementary Table S1. Top identified GO terms for three different categories: biological processes, molecular functions, and cellular components.

Name	# genes (DE/ALL)	FDR p-value
**1. Biological Processes**		
extracellular matrix organization	**59** / 322	1.085×10^{-24}
extracellular structure organization	**59** / 323	1.085×10^{-24}
anatomical structure morphogenesis	**154** / 2149	1.127×10^{-20}
tube development	**88** / 833	1.212×10^{-20}
cell migration	**104** / 1139	3.701×10^{-20}
**2. Molecular Functions**		
collagen binding	**20** / 60	8.775×10^{-13}
extracellular matrix structural constituent	**26** / 128	1.282×10^{-11}
signaling receptor binding	**82** / 1128	1.841×10^{-10}
extracellular matrix binding	**15** / 48	2.711×10^{-9}
protein-containing complex binding	**72** / 1079	1.643×10^{-7}
**3. Cellular components**		
extracellular matrix	**74** / 410	1.000×10^{-24}
extracellular region	**206** / 2945	1.000×10^{-24}
extracellular space	**176** / 2333	1.000×10^{-24}
collagen-containing extracellular matrix	**57** / 318	1.000×10^{-24}
cell surface	**66** / 563	2.077×10^{-18}

Supplementary Table S2. List of the HPE-affected collagen family genes.

Gene	Fold change	FDR p-value
COL5A3	3.51	0.00
COL16A1	1.78	0.00
COL4A1	1.44	$1.63x10^{-10}$
COL5A2	1.44	$5.86x10^{-12}$
COL18A1	1.43	$7.58x10^{-8}$
COL1A1	1.41	$2.47x10^{-11}$
COL3A1	1.41	$6.46x10^{-11}$
COL6A2	1.36	$3.24x10^{-9}$
COL6A1	1.31	$3.89x10^{-7}$
COL1A2	1.21	$1.18x10^{-3}$

Supplementary Table S3. Oligonucleotide primers used for qPCR in this study.

Target gene	Direction	Sequence 5'- 3'
COL1A1	Forward	5′- TCTGCGACAACGGCAAGGTG -3′
	Reverse	5′- GACGCCGGTGGTTTCTTGGT -3′
COL5A3	Forward	5′- CCTGCCTCTATCCCGACAAG -3′
	Reverse	5′- GCGTCCACGTAGGAGAACTTCT -3′
ELN	Forward	5′- CTCCTGCTGTCCATCCTCCA -3′
	Reverse	5′- CGAGACCAGCCCCTGGATAA -3′
HAS2	Forward	5′- GTTGGGGGAGATGTCCAGATTT -3′
	Reverse	5′- TGCACTGAACACACCCAAAA -3′
ITGA11	Forward	5′- TCACGGACACCTTCAACATGG -3′
	Reverse	5′- CCAGCCACTTATTGCCACTGA -3′
TGFBI	Forward	5′- ATCCCAGACTCAGCCAAGAC-3′
	Reverse	5′- GGTCAACCGCTCACTTCCAG -3′
VCAN	Forward	5′- AGGTGGTCTACTTGGGGTGA -3′
	Reverse	5′- TGGTTGTAGCCTCTTTAGGTTT -3′
GAPDH	Forward	5′- GACTCATGACCACAGTCCATGC -3′
	Reverse	5′- AGAGGCAGGGATGATGTTCTG -3′
